# Assessing the reliability and educational value of YouTube videos on computer-controlled local anesthesia in dentistry

**DOI:** 10.1371/journal.pone.0329291

**Published:** 2025-08-07

**Authors:** Hulya Cerci Akcay, Erdal Cem Kargu, Nefise Seker

**Affiliations:** 1 Department of Pedodontics, Kocaeli Health and Technology University, Kocaeli, Turkey; 2 Faculty of Dentistry, Kocaeli Health and Technology University, Kocaeli, Turkey; 3 Department of Public Health, Faculty of Medicine, Ankara University, Ankara, Turkey; International Medical University, MALAYSIA

## Abstract

**Objective:**

This study aimed to assess the scientific accuracy, content quality, and educational value of YouTube™ videos related to computer-controlled local anesthesia (CCLA) techniques in dentistry.

**Materials and methods:**

A total of 100 videos were screened using predefined keywords, and 48 met the inclusion criteria. Videos were assessed using the Global Quality Scale (GQS), DISCERN tool, JAMA benchmark criteria, and the Video Information and Quality Index (VIQI). Scientific content was scored using a structured rubric across six domains. Interobserver reliability was evaluated using Weighted Kappa and Intraclass Correlation Coefficient (ICC). Confidence intervals were calculated for key metrics. Group comparisons were performed using the Mann-Whitney U test, and correlations were analyzed using Spearman’s rho (p < 0.05).

**Results:**

Videos from academic sources had significantly higher scores across all quality and reliability indicators. The mean GQS was 2.6 (95% CI: 2.3–2.9), DISCERN 11.7 (95% CI: 10.8–12.6), JAMA 1.8 (95% CI: 1.7–1.9), and VIQI 12.5 (95% CI: 11.7–13.3). Strong positive correlations were found between DISCERN and VIQI (r = 0.809), and between total content score and both DISCERN (r = 0.803) and VIQI (r = 0.655).

**Conclusion:**

Although YouTube™ provides accessible information on CCLA, many videos lack scientific rigor and educational depth. Content produced by academic institutions is significantly more reliable. Dental educators are encouraged to integrate high-quality video content into curricula to improve media literacy and student learning outcomes.

## Introduction

In today’s digital era, video-sharing platforms such as YouTube™ have become widespread sources of information for both healthcare professionals and the general public. With over 2.7 billion users and 122 million daily viewers, YouTube™ plays a prominent role in shaping health-related perceptions and patient behaviors [1,2]. Numerous studies have shown the growing influence of such platforms on health literacy and decision-making processes [3–8].

Pain, a subjective and multidimensional experience, is a common concern in dental procedures, particularly in pediatric patients. Although local anesthesia is routinely administered to eliminate procedural pain, the injection process itself may trigger anxiety and discomfort due to factors like needle insertion, pressure, and tissue trauma [9–11].

To overcome these challenges, Computer-Controlled Local Anesthesia (CCLA) systems have been introduced. These systems deliver anesthetic agents at a controlled, consistent rate via computer-assisted mechanisms, leading to reduced injection pain and increased patient comfort [12–14]. While CCLA devices (e.g., The Wand, STA system) are not yet universally adopted in all clinical settings, their usage has increased, especially in pediatric and anxiety-prone populations [15,16].

Despite the clinical relevance of CCLA, little is known about how this technology is represented in digital media. YouTube™, as a widely used video platform, hosts vast unregulated health content, including videos related to local anesthesia. However, the scientific quality, accuracy, and educational value of such content remain underexplored. Prior studies on fluoride treatment, dental trauma, and sedation have shown that online videos often suffer from poor structure, insufficient referencing, and low reliability [17–20].

This study aimed to fill this gap by evaluating the reliability and educational quality of YouTube™ videos on computer-controlled local anesthesia (CCLA) in dentistry.

Although the term “digital dental anesthesia” was originally used in the search strategy to reflect common public terminology, the term “Computer-Controlled Local Anesthesia (CCLA)” is used throughout this manuscript to ensure technical accuracy and consistency with current literature.

## Materials and methods

### Video selection and data collection

A systematic search was conducted on the YouTube™ platform (www.youtube.com) on **February 25, 2025**, using a computer located in **Turkey.** The purpose was to identify publicly accessible videos focusing on **computer-controlled local anesthesia (CCLA)** in dentistry. The search terms used included “digital dental anesthesia,” “computer-assisted anesthesia in dentistry,” “painless anesthesia dentistry,” “The Wand dental anesthesia,” and “STA anesthesia dentistry.” These keywords were selected based on both existing literature and public usage trends. The aim was to capture the terminology that actual YouTube users would employ when seeking content about these anesthesia techniques. Before initiating the search, the browser history and cookies were cleared to prevent algorithmic bias. The default “Sort by relevance” setting on YouTube™ was used to retrieve the most contextually aligned videos for each keyword.

The search was performed in one session to limit the impact of time-sensitive algorithm fluctuations, as YouTube™ rankings are known to change based on user interaction and platform activity. Given that prior research suggests users primarily engage with only the **first three pages** of YouTube™ search results, this study followed a similar approach by recording the URLs of the **first 100 videos** retrieved across all keyword queries [18]. This strategy ensured the selection of content most likely to be encountered by the average viewer and maximized the relevance and visibility of the dataset. Additional metadata for each video—including duration, upload date, view count, likes, dislikes, number of comments, and uploader category—was collected and tabulated. Videos were categorized according to their source into four groups: (1) individual dentists, (2) universities or academic institutions, (3) health information platforms, and (4) other individual users.

### Eligibility criteria

**Fig 1:**
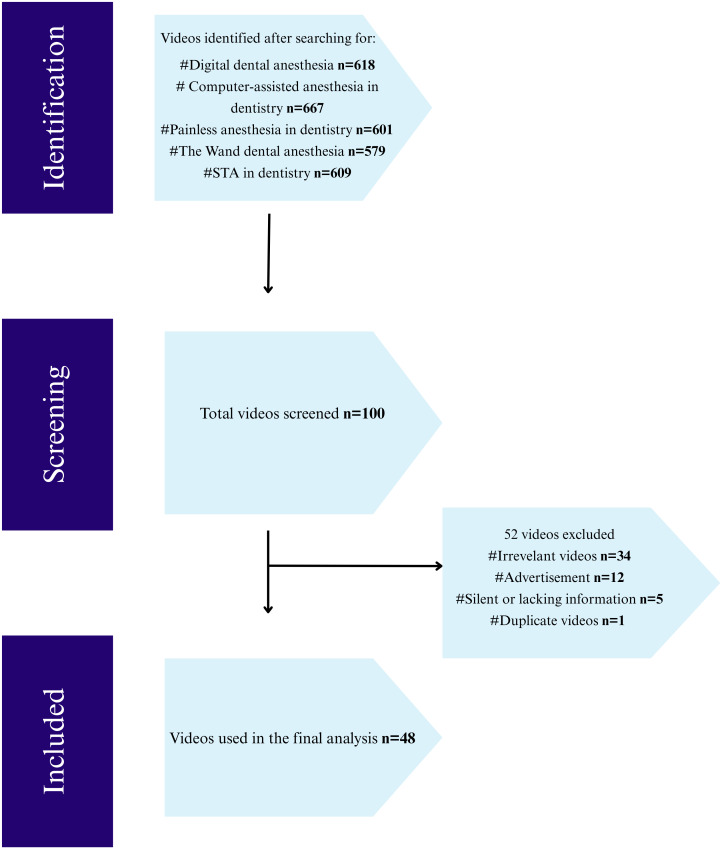
Flowchart for video selection. (100 videos selected based on relevance ranking and expected user behavior (i.e., first three pages of search results)).

To ensure methodological rigor and content consistency, strict inclusion and exclusion criteria were applied, as illustrated in Fig 1, which outlines the video screening and selection process (Fig 1).

Videos were included if they met the following conditions: (1) they were in English, (2) had a minimum resolution of 240 pixels (240p) to ensure basic visual clarity, (3) were less than 30 minutes in duration, and (4) had a primary focus on computer-controlled local anesthesia (CCLA). In addition, the videos needed to contain explanatory content either through narration, subtitles, or visual demonstration to qualify as educational material.

Videos were excluded if they were non-English, of insufficient visual quality, promotional in nature, created for entertainment purposes, or unrelated to CCLA. Duplicate videos and YouTube “shorts” were also excluded due to their limited duration and lack of educational depth. The inclusion and exclusion process was conducted by an independent researcher (NS), who specializes in public health. This ensured objectivity and reduced selection bias in the dataset creation.

After applying all eligibility criteria, the resulting pool of qualified videos was further refined to a final dataset. In accordance with established literature on user interaction with video search platforms, which indicates that viewers predominantly engage with content appearing within the first three pages of search results, the first 100 videos were selected based on YouTube™’s default relevance-based ranking algorithm across all search terms [18]. This sampling strategy was employed to enhance ecological validity by reflecting the content most likely to be encountered by typical users during routine information-seeking behavior.

#### Reliability and educational value assessment of video content.

Each included video was independently evaluated by two trained reviewers: HCA, an experienced pediatric dentist and academic staff member specializing in behavioral dentistry and multimedia education, and ECK**,** a fifth-year dental student with academic interest in computer-assisted anesthesia and digital media analysis. Both reviewers received orientation on the use of all evaluation tools prior to the analysis. Inter-rater disagreements were resolved through structured consensus meetings.

To measure the scientific and educational content, a structured scoring rubric was developed based on prior content evaluation literature. The rubric consisted of six primary domains: (1) definition of CCLA, (2) indications and contraindications, (3) procedural steps and technique demonstration, (4) advantages and disadvantages, (5) comparisons with traditional anesthesia methods, and (6) patient-targeted communication and education. Each domain was scored on a 5-point Likert scale (1 = very poor, 2 = poor, 3 = moderate, 4 = good, 5 = excellent), with a maximum cumulative score of 30 points per video.

In addition to the content-specific rubric, four validated tools were used to assess video quality and reliability. The accuracy and reliability of YouTube™ videos were evaluated based on the criteria set by the Journal of the American Medical Association (JAMA). These criteria assess four essential aspects: authorship, attribution, disclosure, and currency. Each component is awarded 1 point if met, and the total JAMA score is determined by summing the points, providing an overall measure of the video’s credibility [20]. The DISCERN instrument (Quality Criteria for Consumer Health Information) was used to assess the reliability and quality of the videos. This tool comprises three sections with a total of 16 questions. Each question was rated on a 5-point Likert scale, with a maximum possible score of 80. Based on their overall DISCERN score, videos were categorized into five groups: very poor (16–26), poor (27–38), fair (39–50), good (51–62), and excellent (>62) [21]. The overall quality of the videos was evaluated using the Global Quality Scale (GQS), which is designed to assess their general quality. The GQS employs a 5-point rating system, where 1 represents the lowest quality and 5 signifies the highest. Based on their scores, videos were classified as high quality (4 or 5), medium quality (3), or low quality (1 or 2). A score of 1 indicates that the video lacks informative value and is not beneficial for patients, while a score of 2 suggests the presence of limited information but remains of low quality, making it unhelpful. A score of 3 denotes moderate quality with some essential content, whereas a score of 4 reflects good quality with structured information and relevance to patients. Finally, a score of 5 signifies excellent quality, with well-structured information flow and highly useful content for patients [22]. All tools were applied consistently to each video, and each score was recorded independently by both reviewers.

#### Data transfer and statistical analysis.

Data extracted from YouTube™ and the evaluation scores were compiled and analyzed using IBM SPSS Statistics version 29. Normality of the data was assessed using the Shapiro-Wilk test and visual inspection of histograms. Given that the majority of the variables were non-normally distributed, non-parametric statistical tests were applied. The Mann-Whitney U test was used to compare video scores based on upload source (academic vs. individual). All data were collected from publicly available YouTube™ content, and no login, scraping, or third-party data extraction tool was used. The data collection process complied with the platform’s terms of use.

To assess interobserver agreement, Weighted Kappa coefficients were calculated for ordinal scales (GQS, DISCERN), and Intraclass Correlation Coefficients (ICC) were calculated for continuous measures (VIQI, JAMA, total scores), using a two-way mixed-effects model. Agreement thresholds were interpreted as follows: values >0.80 indicated excellent agreement, 0.61–0.80 good, and 0.41–0.60 moderate [23–25].

Although final ratings were determined through consensus in cases of disagreement, interobserver agreement metrics (Weighted Kappa and ICC) were calculated based on the initial independent ratings of both reviewers. This approach enabled the evaluation of initial consistency and objectivity between raters prior to consensus.

Correlations between scoring systems (e.g., DISCERN and VIQI, total score and GQS) were analyzed using Spearman’s rho. In addition to statistical significance (p < 0.05), 95% confidence intervals (CIs) were computed for mean values of central scoring metrics to enhance interpretability and robustness of the findings.

This study used only publicly available data from the YouTube™ platform and did not involve any human or animal participants; therefore, no formal ethical approval was required. However, all ethical principles for conducting research involving digital media sources were strictly followed. The authors affirm that there were no instances of data manipulation, fabrication, or unethical research practices.

## Results

A total of 100 videos were initially screened, from which 48 met the eligibility criteria and were included in the final analysis. As shown in Table 1, the videos had a mean view count of 154.8 ± 221.9 (95% CI: 98.3–211.3), with view numbers ranging from 1.2 to 779. The average number of likes was 48.4 ± 117.1 (95% CI: 16.2–80.6), while dislikes averaged 2.1 ± 8.4 (95% CI: 0.0–4.4). The interaction index, calculated as the ratio of likes and dislikes to total views, was relatively low at 0.5 ± 0.8 (95% CI: 0.3–0.7), indicating limited viewer engagement across most videos.

**Table 1:** Descriptive statistics regarding the general features and content quality scores of.

	Mean ± Sd (95% CI)	Median (Q3–Q1)
**Total Number of Views**	154.8 ± 221.9 (92.0–217.6)	27.4 (232–3.5)
**Total Number of Likes**	48.4 ± 117.1 (15.2–81.6)	8 (27.5–1)
**Total Number of Dislikes**	2.1 ± 8.4 (0–4.5)	0 (0–0)
**Interaction Index**	0.5 ± 0.8 (0.28–0.73)	0.4 (0.67–0.195)
**Definition of digital anaesthesia**	2.6 ± 1.1 (2.29–2.91)	2.5 (3–2)
**Indications/Contraindications**	2.3 ± 1 (2.02–2.58)	2 (3–1)
**Implementation stages and technical details**	3.2 ± 1.3 (2.83–3.57)	3 (4–2)
**Advantages/Disadvantages**	3 ± 1 (2.72–3.28)	3 (4–2)
**Comparison with alternative anaesthesia techniques**	2.7 ± 1 (2.42–2.98)	3 (3–2)
**Follow-up/Complications**	2 ± 1 (1.72–2.28)	2 (3–1)
**Information towards patients**	2.2 ± 1.1 (1.89–2.51)	2 (3–1)
**Total Score**	18 ± 5.9 (16.33–19.67)	18 (22.5–13)

In terms of scientific and educational content, the mean score for definition of CCLA was 2.6 ± 1.1 (95% CI: 2.3–2.9), while indications/contraindications scored 2.3 ± 1.0 (95% CI: 2.0–2.6). The implementation and technical detail domain received the highest mean score (3.2 ± 1.3, 95% CI: 2.8–3.6), whereas follow-up/complication information received the lowest (2.0 ± 1.0, 95% CI: 1.7–2.3). The mean total content score across the six domains was 18.0 ± 5.9 (95% CI: 16.1–19.9) (Table 1).

As detailed in Table 2, the Global Quality Scale (GQS) yielded a mean score of 2.6 ± 1.1 (95% CI: 2.3–2.9), suggesting that most videos were of low to moderate quality. The DISCERN score, reflecting content reliability, averaged 11.7 ± 4.2 (95% CI: 10.5–12.9), placing the majority of videos in the “very poor” range. JAMA benchmark scores had a mean of 1.8 ± 0.6 (95% CI: 1.6–2.0), highlighting a lack of transparency and proper attribution. The mean Video Information and Quality Index (VIQI) was 12.5 ± 3.6 (95% CI: 11.4–13.6), indicating moderate structure and audiovisual coherence.

**Table 2:** Descriptive statistics for video characteristics, content quality domains, and evaluation scores (GQS, DISCERN, JAMA Benchmark, VIQI).

	Mean ± Sd (95% CI)	Median (Q3–Q1)
**Global Quality Scale**	2.6 ± 1.1 (2.29–2.91)	2 (3–2)
**DISCERN Skoru**	11.7 ± 4.2 (10.51–12.89)	11 (14–9)
**JAMA**	1.8 ± 0.6 (1.63–1.97)	2 (2–1)
**Video Information and Quality Index**	12.5 ± 3.6 (11.48–13.52)	13 (15–9)

Interobserver agreement was evaluated to ensure rating consistency. As presented in Table 3, Weighted Kappa values showed moderate agreement for GQS (κ = 0.599) and good agreement for DISCERN (κ = 0.783, p < 0.001). The intraclass correlation coefficients for JAMA (ICC = 0.921, 95% CI: 0.871–0.954) and VIQI (ICC = 0.915, 95% CI: 0.864–0.951) demonstrated excellent inter-rater reliability. The composite total score, defined as the sum of DISCERN, JAMA, and VIQI scores, achieved the highest level of agreement with an ICC of 0.965 (95% CI: 0.938–0.980), reflecting strong alignment between evaluators across all scales.

**Table 3:** Interobserver agreement levels for video evaluation scales using Weighted Kappa and Intraclass Correlation Coefficient (ICC). Note: Weighted Kappa was used for the Global Quality Scale and DISCERN score, while ICC (Two-Way Mixed Effects ICC) was applied for the other scales.

	ICC/Weighted Kappa	%95 CI	P Value
**Global Quality Scale (Weighted Kappa)**	0.599	(0.431–0.768)	<0.001
**DISCERN Skoru (Weighted Kappa)**	0.783	(0.678–0.888)	<0.001
**JAMA (ICC)**	0.921	(0.864–0.955)	<0.001
**Video Information and Quality Index (ICC)**	0.915	(0.853–0.951)	<0.001
**Total Score (ICC)**	0.965	(0.938–0.980)	<0.001

As shown in Table 4, videos uploaded by academic institutions or health-related websites outperformed those created by individual dentists across all measured parameters. DISCERN scores were significantly higher in academic videos (median: 14 vs. 9, p < 0.001), as were VIQI (15 vs. 10, p < 0.001) and GQS scores (3 vs. 2, p = 0.023). Furthermore, these videos provided more comprehensive definitions of CCLA (p = 0.046), better explanations of clinical indications (p = 0.028), and clearer implementation details (p = 0.006). Academically produced content also outperformed in patient information (p = 0.043) and received significantly more likes (median: 11.5 vs. 2, p = 0.003), suggesting that viewer appreciation may be linked to higher scientific quality.

**Table 4:** Comparison of scientific quality and content according to video sources.

	Mean ± Sd	Median (Q3–Q1)	Confidence Interval
**Total Number of Views**	154.8 ± 221.9	27.4 (232–3.5)	154.8 (92.0–217.6)
**Total Number of Like**	48.4 ± 117.1	8 (27.5–1)	48.4 (15.2–81.6)
**Total Number of Dislike**	2.1 ± 8.4	0 (0–0)	2.1 (0–4.5)
**Interaction Index**	0.5 ± 0.8	0.4 (0.67–0.195)	0.5 (0.28–0.73)
**Definition of digital anaesthesia**	2.6 ± 1.1	2.5 (3–2)	2.6 (2.29–2.91)
**Indications/Contraindications**	2.3 ± 1	2 (3–1)	2.3 (2.02–2.58)
**Implementation stages and technical details**	3.2 ± 1.3	3 (4–2)	3.2 (2.83–3.57)
**Advantages/Disadvantages**	3 ± 1	3 (4–2)	3.0 (2.72–3.28)
**Comparison with alternative anaesthesia techniques**	2.7 ± 1	3 (3–2)	2.7 (2.42–2.98)
**Follow-up/Complications**	2 ± 1	2 (3–1)	2.0 (1.72–2.28)
**Information towards patients**	2.2 ± 1.1	2 (3–1)	2.2 (1.89–2.51)
**Total Puan**	18 ± 5.9	18 (22.5–13)	18.0 (16.33–19.67)

Correlation analysis, summarized in Table 5, revealed strong positive relationships between several key scoring systems. VIQI was strongly correlated with GQS (r = 0.702, p < 0.001), and even more strongly with DISCERN (r = 0.809, p < 0.001), underscoring the link between structural quality and informational accuracy. The composite total score also correlated strongly with DISCERN (r = 0.803, p < 0.001) and moderately with VIQI (r = 0.655, p < 0.001). Additionally, JAMA scores showed a moderate correlation with VIQI (r = 0.468, p = 0.001), suggesting that transparency and authorship information enhance perceived content quality. Spearman correlation test was used. *p < 0.05, **p < 0.01 were considered statistically significant.**

**Table 5:** Correlation analysis among scientific quality scales.

		Total Number of Views	Total Number of Likes	Total Number of Dislikes	Interaction Index	Global Quality Scale	DISCERN	jAMA	Video Information and Quality Index
**Total Number of Likes**	R	−0.320*							
	p Value	0.026							
**Total Number of Dislikes**	R	0.078	.434**						
	p Value	0.600	0.002						
**Interaction Index**	R	−0.054	.510**	−0.166					
	p Value	0.713	<0.001	0.259					
**Global Quality Scale**	R	−0.187	.349*	0.249	0.157				
	p Value	0.203	0.015	0.088	0.287				
**DISCERN**	R	−0.215	.458**	0.215	0.237	.783**			
	p Value	0.143	0.001	0.142	0.105	<0.001			
**jAMA**	R	−0.092	0.165	−0.032	0.021	.609**	.494**		
	p Value	0.535	0.262	0.831	0.888	<0.001	<0.001		
**Video Information and Quality Index**	R	−0.237	.341*	0.106	0.202	.702**	.809**	.468**	
	P Value	0.105	0.018	0.472	0.168	<0.001	<0.001	0.001	
**Total Score**	R	−0.200	0.236	0.136	0.136	.792**	.803**	.492**	.655**
	p Value	0.173	0.107	0.357	0.357	<0.001	<0.001	<0.001	<0.001

Spearman correlation test was used. *p < 0.05, p < 0.01 were considered statistically significant.

## Discussion

Effective pain control plays a fundamental role in the success of dental procedures, especially for patients who are young or highly anxious [26,27]. In recent years, computer-controlled local anesthesia (CCLA) systems have emerged as promising alternatives to conventional syringe techniques, offering improved comfort, greater precision, and enhanced cooperation [28–30]. Despite this growing clinical relevance, there has been a striking lack of investigation into how CCLA is represented and understood in publicly accessible digital platforms [10,30–32]. The present study provides the first comprehensive evaluation of the reliability, scientific quality, and educational value of YouTube™ videos focused on CCLA in dentistry.

The findings indicate that while a number of videos achieve moderate to high scores in technical presentation, overall scientific content and reliability remain suboptimal. The mean DISCERN score (11.7 ± 4.2) and Global Quality Scale (GQS) score (2.6 ± 1.1) suggest that the overall reliability and educational quality of the videos were low. Similarly, the JAMA benchmark score (1.8 ± 0.6), reflecting source attribution and scientific transparency, was notably insufficient. A significant proportion of the evaluated content was characterized by incomplete explanations, lack of references, and minimal disclosure of authorship or production background. These results align with prior studies on YouTube™ content in dentistry, including topics such as fluoride therapy, teething, and local anesthesia, which have similarly reported inconsistent accuracy and a dominance of low-quality content [29,33–35].

A particularly noteworthy observation in this study is the disconnect between video popularity and content quality. The Video Information and Quality Index (VIQI) score (12.5 ± 3.6) observed in our study is comparable to prior findings on videos related to dental anesthesia and fluoride therapy (VIQI ≈ 14), indicating a recurrent deficiency in structural coherence and informational depth across dental-related content on YouTube™ [29,33]. Notably, strong positive correlations were found between VIQI and other quality measures, including GQS (r = 0.702, p < 0.001) and DISCERN (r = 0.809, p < 0.001), suggesting that videos with higher structural quality tend to be perceived as more reliable and informative. However, despite this, videos with lower DISCERN and VIQI scores were often found to have higher view counts, echoing similar findings in prior research on dental caries, oral hygiene, and orthodontics [14,36,37]. This highlights a core weakness in the platform’s content delivery model, which tends to amplify engagement-based metrics rather than informational accuracy. Therefore, patients and even students may be disproportionately exposed to visually appealing but scientifically unreliable content.

One of the most significant contributions of this study lies in its comparative analysis based on the source of video upload. Videos uploaded by academic institutions or health-related websites consistently scored higher across all quality measures, including DISCERN, GQS, VIQI, and content subcategories such as definition, indications, and patient communication. This is consistent with findings from systematic reviews and individual studies indicating that videos produced by healthcare professionals or academic bodies offer more evidence-based, structured, and ethically grounded information [14,37,38]. Interestingly, our study found that videos with lower content scores had significantly higher view counts, a pattern that mirrors findings from studies on nutrition, dental caries and teething content on YouTube™ [39,40]. This discrepancy between viewer engagement and content quality underlines a key limitation of using popularity metrics—such as views and likes—as proxies for educational value. Therefore, without content regulation or quality assurance mechanisms, patients and caregivers may be disproportionately exposed to visually appealing but scientifically inadequate content.

The strong correlation found between the total content score and DISCERN (r = 0.803, p < 0.001) further supports the validity of DISCERN as a reliable tool for assessing the overall scientific integrity of video content. Moreover, a moderate correlation between the JAMA benchmark and VIQI (r = 0.468, p = 0.001) suggests that transparency and scientific referencing are also associated with better content structure and accuracy. Taken together, these results reinforce the importance of institutional presence and evidence-based frameworks in shaping reliable digital health communication.

### Strengths and limitations

One of the key strengths of this study is its comparative approach, which evaluated YouTube™ videos based on their source of upload. Findings demonstrated that videos produced by academic institutions or health-related organizations consistently scored higher across all quality metrics—including DISCERN, GQS, VIQI, and subdomains such as definition, indications, and patient communication—than those produced by individual users. This reinforces conclusions from earlier systematic reviews showing that institutionally produced health-related content tends to be more reliable, structured, and ethically grounded.

However, several limitations should be acknowledged. First, although pediatric relevance was briefly noted in the introduction, the study’s methodology did not focus specifically on pediatric-oriented content. Therefore, the results should be interpreted in the context of general dental education rather than pediatric practice. Second, the dataset was limited to English-language videos, excluding potentially informative content in other languages. Third, due to the dynamic nature of the YouTube™ platform, view counts, likes, and rankings change over time, which restricts the temporal generalizability of the findings. Fourth, no demographic data were available for viewers, limiting the ability to assess content impact on specific audiences. Lastly, although validated tools were used, some degree of subjectivity in scoring remains inherent to human evaluation.

### Implications and future directions

The results of this study hold important implications for both digital health communication and dental education. Given the consistent finding that videos from academic sources outperform others, it is clear that **institutional engagement is essential** in creating and curating trustworthy educational content. Academic contributors are more likely to follow structured, evidence-based communication strategies, including use of visual supports, accurate terminology, and references to credible sources.

To mitigate the risk of public exposure to low-quality health information, **platform-level interventions** could be considered. YouTube™ might collaborate with academic bodies and professional organizations to implement **algorithmic filters, verification badges,** or **educational metadata tags** that prioritize scientifically credible content. Additionally, peer-review indicators or institutional endorsements could be embedded in video metadata to help users identify trustworthy materials more efficiently.

From an educational standpoint, the findings underscore the need for **dental educators to incorporate validated video resources** into formal curricula. This could not only enhance student understanding of advanced clinical tools like CCLA, but also foster **critical media literacy**, allowing students to evaluate online content independently and responsibly. Future research may explore similar analyses across different languages, platforms (e.g., TikTok, Instagram), or topic areas to further assess the role of social media in shaping dental knowledge dissemination.

## Conclusion

This study reveals critical gaps in the reliability and educational value of YouTube™ videos related to computer-controlled local anesthesia in dentistry. While academic and institutionally affiliated content demonstrated higher quality across all domains, a majority of publicly available videos lacked scientific rigor, transparency, and comprehensive information. To address this gap, dental educators are strongly encouraged to integrate high-quality, evidence-based video content into formal curricula. Such efforts may not only enhance learning outcomes, but also empower students to navigate and critically evaluate digital health information, thereby fostering a generation of clinicians who are both clinically competent and media literate.

## Supporting information

S1 File Raw dataset of YouTube video evaluation related to computer-controlled dental anesthesia.  (XLSX)

## References

[1] Apel Z, Fagundes NCF, Sharmin N, Nassar U, Gow G, Apel D, et al. Social media in oral health education: a scoping review. Eur J Dent Educ. 2025;29(1):50–63. doi: 10.1111/eje.13053 39462438 PMC11729249

[2] Global Media Insight. YouTube users statistics 2025 [Internet]. 2025 [cited 2025 Mar 23]. Available from: https://www.globalmediainsight.com/blog/youtube-users-statistics/#YouTube_Users_Statistics_2025_Infographics

[3] Aguirre PEA, Anibal I, Lotto M, Strieder AP, Cruvinel T. Decoding early childhood caries: an in-depth analysis of YouTube videos for effective parental education. Eur Arch Paediatr Dent. 2023;24(6):701–9. doi: 10.1007/s40368-023-00830-1 37610682

[4] Acosta JM, Detsomboonrat P, Pisarnturakit PP, Urwannachotima N. The use of social media on enhancing dental care and practice among dental professionals: cross-sectional survey study. JMIR Form Res. 2025;9:e66121. doi: 10.2196/66121 39757575 PMC11723565

[5] Madathil KC, Rivera-Rodriguez AJ, Greenstein JS, Gramopadhye AK. Healthcare information on YouTube: a systematic review. Health Inform J. 2015;21(3):173–94. doi: 10.1177/1460458213512220 24670899

[6] Househ M. The use of social media in healthcare: organizational, clinical, and patient perspectives. Stud Health Technol Inform. IOS Press; 2013. doi: 10.3233/978-1-61499-203-5-244 23388291

[7] Wong NSM, Yeung AWK, McGrath CP, Leung YY. Qualitative evaluation of YouTube videos on dental fear, anxiety and phobia. Int J Environ Res Public Health. 2022;20(1):750. doi: 10.3390/ijerph20010750 36613071 PMC9819845

[8] Monteiro CMG, Silva KS, Tavares FOM, Dias M de O, Maia LC, Pithon MM. Assessment of the reliability of YouTube™ videos about zirconia crowns in pediatric dentistry. Eur Arch Paediatr Dent. 2023;24(5):585–90. doi: 10.1007/s40368-023-00822-1 37501012

[9] Gosnell ES, Thikkurissy S. Assessment and Management of Pain in the Pediatric Patient. Pediatric Dentistry. 1st ed. Philadelphia: Elsevier; 2019. pp. 97–115.e1. doi: 10.1016/b978-0-323-60826-8.00007-9

[10] Berrendero S, Hriptulova O, Salido MP, Martínez-Rus F, Pradíes G. “Comparative study of conventional anesthesia technique versus computerized system anesthesia: a randomized clinical trial”. Clin Oral Investig. 2021;25(4):2307–15. doi: 10.1007/s00784-020-03553-5 32862249

[11] Vishwanathaiah S, Maganur PC, Al-Shomrani YK, Alhijji HH, Koriri AYA, Mergami JMM, et al. Evaluation of the quality of educational content of YouTube videos on silver diamine fluoride. Int J Clin Pediatr Dent. 2024;17(12):1399–403. doi: 10.5005/jp-journals-10005-3014 39867110 PMC11760415

[12] Kwak E-J, Pang N-S, Cho J-H, Jung B-Y, Kim K-D, Park W. Computer-controlled local anesthetic delivery for painless anesthesia: a literature review. J Dent Anesth Pain Med. 2016;16(2):81–8. doi: 10.17245/jdapm.2016.16.2.81 28879299 PMC5564086

[13] Grace EG, Barnes DM, Reid BC, Flores M, George DL. Computerized local dental anesthetic systems: patient and dentist satisfaction. J Dent. 2003;31(1):9–12. doi: 10.1016/s0300-5712(02)00130-6 12615014

[14] Naik S, Al-Kheraif AA, Vellappally S. Artificial intelligence in dentistry: assessing the informational quality of YouTube videos. PLoS One. 2025;20(1):e0316635. doi: 10.1371/journal.pone.0316635 39746083 PMC11695022

[15] Janik K, Niemczyk W, Peterek R, Rój R, Balicz A, Morawiec T. Computer-controlled local anaesthesia delivery efficacy - a literature review. Saudi Dent J. 2024;36(8):1066–71. doi: 10.1016/j.sdentj.2024.05.012 39176166 PMC11338014

[16] Altuhafy M, Sodhi GS, Khan J. Efficacy of computer-controlled local anesthesia delivery system on pain in dental anesthesia: a systematic review of randomized clinical trials. J Dent Anesth Pain Med. 2024;24(4):245–64. doi: 10.17245/jdapm.2024.24.4.245 39118810 PMC11304040

[17] Ozdemir ZM, Yavuz SA, Gursel Surmelioglu D. YouTube as an information source in deep margin elevation: reliability, accuracy and quality analysis. PLoS One. 2025;20(2):e0318568. doi: 10.1371/journal.pone.0318568 39919079 PMC11805417

[18] Yang S, Brossard D, Scheufele DA, Xenos MA. The science of YouTube: what factors influence user engagement with online science videos? PLoS One. 2022;17(5):e0267697. doi: 10.1371/journal.pone.0267697 35613095 PMC9132274

[19] Lena Y, Dindaroğlu F. Lingual orthodontic treatment: a YouTube™ video analysis. Angle Orthod. 2018;88(2):208–14. doi: 10.2319/090717-602.1 29257704 PMC8312536

[20] Zhang S, Fukunaga T, Oka S, Orita H, Kaji S, Yube Y, et al. Concerns of quality, utility, and reliability of laparoscopic gastrectomy for gastric cancer in public video sharing platform. Ann Transl Med. 2020;8(5):196. doi: 10.21037/atm.2020.01.78 32309343 PMC7154475

[21] Charnock D. The DISCERN Handbook: Quality criteria for consumer health information on treatment choices. Radcliffe: University of Oxford and The British Library; 1998. pp. 7–51.

[22] Loureiro JM, Chaves VCV, Risso PA, Magno MB, Maia LC, de Pithon MM. YouTube™ as a source of tooth avulsion information: a video analysis study. Dent Traumatol. 2023;39(6):616–24. doi: 10.1111/edt.12873 37638632

[23] McHugh ML. Interrater reliability: the kappa statistic. Biochem Med. 2012:276–82. doi: 10.11613/bm.2012.031 PMC3900052 23092060

[24] Koo TK, Li MY. A guideline of selecting and reporting intraclass correlation coefficients for reliability research. J Chiropr Med. 2016;15(2):155–63. doi: 10.1016/j.jcm.2016.02.012 27330520 PMC4913118

[25] Schober P, Boer C, Schwarte LA. Correlation coefficients: appropriate use and interpretation. Anesth Analg. 2018;126(5):1763–8. doi: 10.1213/ANE.0000000000002864 29481436

[26] Kim E-J, Kim HY, Ahn J-H. Neurotoxicity of local anesthetics in dentistry. J Dent Anesth Pain Med. 2020;20(2):55–61. doi: 10.17245/jdapm.2020.20.2.55 32395610 PMC7193059

[27] Esmaeili H, Malekzadeh M, Esmaeili D, Nikeghbal F. Dental anxiety and the effectiveness of local anesthesia. Braz J Oral Sci. 2020;19:e208127. doi: 10.20396/bjos.v19i0.8658127

[28] Carugo N, Paglia L, Re D. Pain perception using a computer-controlled anaesthetic delivery system in paediatric dentistry: a review. Eur J Paediatr Dent. 2020;21(3):180–2. doi: 10.23804/ejpd.2020.21.03.03 32893647

[29] Tozar KN, Erkmen Almaz M. Evaluation of the content of YouTube™ videos about local anesthesia in pediatric dentistry. Selcuk Dent J. 2023;10(2):377–81. doi: 10.15311/selcukdentj.1133063

[30] Pielech M, Sawicki CM. Provider perspectives on pain management practices and needs in pediatric dentistry. J Am Dent Assoc. 2023;154(12):1067–76. doi: 10.1016/j.adaj.2023.09.003 37877929 PMC11078527

[31] de França AJB, da Barbirato DS, de Vasconcellos RJH, Pellizzer EP, de Moraes SLD, do Vasconcelos BCE. Do computerized delivery systems promote less pain and anxiety compared to traditional local anesthesia in dental procedures? A systematic review of the literature. J Oral Maxillofac Surg. 2022;80(4):620–32. doi: 10.1016/j.joms.2021.11.018 34942152

[32] Arabulan S, Önçağ Ö. Computer-controlled local anesthesia in pediatric dentistry. EÜ Dişhek Fak Derg. 2023;44(2):181–6. doi: 10.5505/eudfd.2023.37097

[33] Çağırır Dindaroğlu F, Akan B. YouTube as a source of information for fluoride treatment: a content and quality analysis. EÜ Dişhek Fak Derg. 2022;43(1):53–9. doi: 10.5505/eudfd.2022.50490

[34] Varghese NS, Cherian JM, Thomas AM. Credibility of YouTube™ videos on root canal treatment in children. J Indian Soc Pedod Prev Dent. 2022;40(2):154–8. doi: 10.4103/jisppd.jisppd_171_22 35859407

[35] Topsakal KG, Aksoy M. Analysis of YouTube™ videos related to dental sedation: a cross-sectional study. Turkiye Klinikleri J Dental Sci. 2022;28(3):591–600. doi: 10.5336/dentalsci.2021-86949

[36] Kodonas K, Fardi A. YouTube as a source of information about pulpotomy and pulp capping: a cross sectional reliability analysis. Restor Dent Endod. 2021;46(3):e40. doi: 10.5395/rde.2021.46.e40 34513646 PMC8411001

[37] Kiraz G, Mumcu AK, Kurnaz S. YouTube as a source of information about rubber dam: quality and content analysis. Restor Dent Endod. 2024;49(1):e10. doi: 10.5395/rde.2024.49.e10 38449493 PMC10912544

[38] Uzel İ, Ghabchi B, Akalın A, Eden E. YouTube as an information source in paediatric dentistry education: reliability and quality analysis. PLoS One. 2023;18(3):e0283300. doi: 10.1371/journal.pone.0283300 36961800 PMC10038246

[39] Long M, Forbes LE, Papagerakis P, Lieffers JRL. YouTube videos on nutrition and dental caries: content analysis. JMIR Infodemiol. 2023;3:e40003. doi: 10.2196/40003 37561564 PMC10450531

[40] Eroğlu Çakmakoğlu E, Bakır M. Evaluation of Youtube videos about teething. Akd Med J. 2024. doi: 10.53394/akd.1310739

